# The Role of Neurotransmitters in Protection against Amyloid-****β**** Toxicity by KiSS-1 Overexpression in SH-SY5Y Neurons

**DOI:** 10.1155/2013/253210

**Published:** 2013-07-17

**Authors:** Amrutha Chilumuri, Nathaniel G. N. Milton

**Affiliations:** ^1^Department of Human and Health Sciences, School of Life Sciences, University of Westminster, 115 New Cavendish Street, London W1W 6UW, UK; ^2^Health Sciences Research Centre, University of Roehampton, Holybourne Avenue, London SW15 4JD, UK

## Abstract

Recent studies have suggested that the kisspeptin (KP) and kissorphin (KSO) peptides have neuroprotective actions against the Alzheimer's amyloid-**β** (A**β**) peptide. Overexpression of the human KiSS-1 gene that codes for KP and KSO peptides in SH-SY5Y neurons has also been shown to inhibit A**β** neurotoxicity. The *in vivo* actions of KP include activation of neuroendocrine and neurotransmitter systems. The present study used antagonists of KP, neuropeptide FF (NPFF), opioids, oxytocin, estrogen, adrenergic, cholinergic, dopaminergic, serotonergic, and **γ**-aminobutyric acid (GABA) receptors plus inhibitors of catalase, cyclooxygenase, nitric oxide synthase, and the mitogen activated protein kinase cascade to characterize the KiSS-1 gene overexpression neuroprotection against A**β** cell model. The results showed that KiSS-1 overexpression is neuroprotective against A**β** and the action appears to involve the KP or KSO peptide products of KiSS-1 processing. The mechanism of neuroprotection does not involve the activation of the KP or NPFF receptors. Opioids play a role in the toxicity of A**β** in the KiSS-1 overexpression system and opioid antagonists naloxone or naltrexone inhibited A**β** toxicity. The mechanism of KiSS-1 overexpression induced protection against A**β** appears to have an oxytocin plus a cyclooxygenase dependent component, with the oxytocin antagonist atosiban and the cyclooxygenase inhibitor SC-560 both enhancing the toxicity of A**β**.

## 1. Introduction

Recent studies have suggested that the kisspeptin (KP) and kissorphin (KSO) peptide derivatives of the metastasis-suppressor KiSS-1 gene may have neuroprotective actions against the Alzheimer's amyloid-*β* (A*β*) peptide [1]. The studies have also suggested that stable overexpression of the KiSS-1 gene in SH-SY5Y neurons creates a cell line that is resistant to the neurotoxicity of A*β* [1]. The primary role of KP peptides is as a regulator of hypothalamic-pituitary-gonadal- (HPG-) axis via stimulation of gonadotrophin-releasing hormone (GnRH) release [2]. The KP peptides are ligands for the GPR-54 receptor [3–7] and the neuropeptide FF (NPFF) receptors, NPFFR1 (GPR-147) and NPFFR2 (GPR-74) [3, 4, 6–9]. The KSO peptides have been suggested to be ligands for the NPFF receptors but not the GPR-54 receptor [10]. Both KP and KSO peptides are protective against the A*β* peptide *in vitro* [1]. However, the neuroprotective actions of KP and KSO peptides have been suggested not to be mediated via actions on GPR-54 or NPFF receptors [1]. Fibrillar A*β* peptides stimulate the release of KP peptides [1, 11] and KP has been suggested to colocalize with A*β* deposits in the Alzheimer's brain [11].

The actions of KP peptides are thought to be mediated via activation of either GPR-54 or NPFF receptors. However, *in vivo* actions on the opioid system [12, 13], oxytocin/vasopressin systems [4, 14, 15], neurotransmitter systems [16, 17], activation of endogenous antioxidants [18], activation of nitric oxide [17], and possible activation of prostaglandin synthesis [19] have not been tested with GPR-54 or NPFF receptor antagonists.

The present study was conducted to characterize a model of KiSS-1 gene overexpression neuroprotection against A*β* in SH-SY5Y neurons *in vitro* [1] and to determine the role of neurotransmitter systems in the neuroprotection. The effects of antagonists of KP, NPFF, opioids, oxytocin, estrogen, adrenergic, cholinergic, dopaminergic, serotonergic, and *γ*-aminobutyric acid (GABA) receptors were tested. Inhibitors of catalase, cyclooxygenase, nitric oxide synthase, and the mitogen activated protein kinase cascade were also tested.

## 2. Materials and Methods

### 2.1. Materials

Synthetic A*β* peptides plus anti-kisspeptin antibody were obtained from Bachem. Human SH-SY5Y neuroblastoma cell line was obtained from the Health Protection Agency Cell Culture Collection. ASCAT peptide was obtained from Insight Biotechnology Ltd. 3-Amino-1,2,4-triazole, atosiban, atropine sulphate, 1(S),9(R)-(−)-bicuculline methiodide, BTA-EG4 hydrate, cyproheptadine hydrochloride, DAPT, haloperidol, KP234, mecamylamine hydrochloride, methysergide maleate, naltrexone, N^G^-Methyl-L-arginine acetate salt, PD98059, phenoxybenzamine hydrochloride, prazosin hydrochloride, propranolol hydrochloride, RF9, SC-560, tamoxifen, and yohimbine hydrochloride, plus all other chemicals, were obtained from Sigma-Aldrich.

### 2.2. A*β* Fibril Formation

Batches of synthetic A*β* 1–40 or A*β* 25–35 were dissolved in distilled water at a concentration of 1.0 mg/mL and incubated at 37°C for 24 h, with constant oscillation. Following incubation, the formation of fibrils was confirmed by TEM or Congo red assay as previously described by Milton and Harris [20–22].

### 2.3. Cell Cultures and KiSS-1 Overexpression

Human SH-SY5Y neuroblastoma cells were routinely grown in a 5% CO_2_ humidified incubator at 37°C in a 1 : 1 mixture of Dulbecco's modified Eagle's medium and HAM's F12 with Glutamax (Invitrogen) supplemented with 10% fetal calf serum (FCS), 1% nonessential amino acids, penicillin (100 units/mL), and streptomycin (100 mg/mL) [23]. The human KiSS-1 cDNA clone (NM_002256) was obtained from Origene and PCR cloned into the pcDNA4/TO/myc-His expression vector using forward (5′-TTAGGATCCATGAACTCACTGGTTTCTTGGCA-3′) and reverse (5′-ATACTCGAGGCCCCGCCCAGCGCTTCT-3′) oligonucleotides to create the PKiSS expression vector. SH-SY5Y cells were transfected with PKiSS or control vector using lipofectamine (Invitrogen), and stably expressing clones were selected by culturing in 100 *μ*g/mL Zeocin (Invitrogen). The presence of KiSS-1 overexpression was confirmed by immunocytochemistry and RT-PCR analysis. Human neuroblastoma SH-SY5Y, PKiSS, and PVect cells were cultured in 96-well plates and differentiated with retinoic acid for 7 days prior to experimentation.

### 2.4. Immunocytochemistry

Cells were washed with PBS, fixed with 4% paraformaldehyde for 15 min, and permeabilized in ice cold methanol for 30 min. Cells were incubated in block solution (10% bovine serum albumin in PBS) for 15 min, followed by incubation with primary antibody anti-KP 45–54 (1 : 1000) in block solution for 1 h. Primary antibody was removed followed by 3 × 5 min washes in PBS, prior to incubation with goat anti-rabbit IgG-Alexa-fluor 488 secondary (Abcam PLC, Cambridge; 1 : 500) in block solution for 45 min. Secondary antibody was removed and cells were washed 3 times in PBS. Cells were incubated with 100 *μ*g/mL RNase A for 20 min at 37°C, followed by 3 × 5 min washes and incubation with 1 *μ*M TO-PRO-3 Iodide (642/661; Invitrogen) for 20 min. Cells were washed 3 times in PBS and fluorescence was visualized by sequential scanning using a Leica TCS SP2 confocal system (Leica Microsystems, Milton Keynes, UK) [11].

### 2.5. Western Blotting of Conditioned Media

To determine the presence of KP released into the media from KiSS-1-overexpressing and vector control cells proteins were purified from 6 mls of conditioned media using an Amicon system (Merck Millipore UK). Proteins in extracts were resuspended in sample buffer before boiling for 5 min and separation of samples using a 15% SDS-PAGE gel. Proteins were then transferred to a nitrocellulose membrane and membranes were blocked with 3% nonfat dried milk powder in PBS containing 0.1% Tween 20 (1 h at room temperature). Membranes were incubated overnight at 4°C with rabbit anti-KP 45−54 antibody. Unbound antibody was rinsed from the membranes before incubation with horseradish peroxidase-conjugated goat anti-rabbit secondary antibody. Immunoreactivity was detected using an enhanced chemiluminescence substrate and UVP BioImaging system.

### 2.6. Reverse Transcription Polymerase Chain Reaction (RT-PCR)

To determine the steady-state levels of KiSS-1 mRNA, total RNA was isolated from KiSS-1-overexpressing and vector control cells using a Qiagen RNeasy extraction kit (Cat No: 74104) according to the manufacturer's instructions. RT-PCR was performed using the Qiagen one-step RT-PCR reagent kit (Cat. no: 210210) with KiSS-1 forward 5′-TTAGGATCCATGAACTCACTGGTTTCTTGGCA-3′ and reverse (5′-ATACTCGAGGCCCCGCCCAGCGCTTCT-3′) primers. The level of *β*-actin was used to normalize loadings of total RNA [4].

### 2.7. Effects of Neurotransmitter Antagonists

Test drugs were used at the following concentrations: anti-KP (10 *μ*g/mL); KP234 (10 *μ*M); RF9 (10 *μ*M); ASCAT (100 *μ*M); BTA-EG4 hydrate (10 *μ*M); naloxone (1 *μ*M); naltrexone (1 *μ*M); atosiban (1 *μ*M); phenoxybenzamine hydrochloride (10 *μ*M); prazosin hydrochloride (250 nM); yohimbine hydrochloride (50 nM); propranolol hydrochloride (50 nM); atropine sulphate (10 *μ*M); mecamylamine hydrochloride (10 *μ*M); haloperidol (10 *μ*M); cyproheptadine hydrochloride (10 nM); methysergide maleate (1 *μ*M); 1(S),9(R)-(−)-bicuculline methiodide (50 *μ*M); tamoxifen (10 *μ*M); 3-Amino-1,2,4-triazole (50 mM); SC-560 (1 *μ*M); N^G^-Methyl-L-arginine acetate salt (1 mM) and PD98059 (50 *μ*M). Stock solutions of at least 100x maximum required concentration for testing were prepared in PBS (anti-KP), ddH_2_O (KP234, RF9, ASCAT, naloxone, naltrexone, atosiban, yohimbine hydrochloride, 1(S),9(R)-(−)-bicuculline methiodide, 3-Amino-1,2,4-triazole, N^G^-Methyl-L-arginine acetate salt), methanol (phenoxybenzamine hydrochloride, prazosin hydrochloride), ethanol (atropine sulphate, mecamylamine hydrochloride, cyproheptadine hydrochloride), or DMSO (BTA-EG4 hydrate, propranolol hydrochloride, haloperidol, methysergide maleate, tamoxifen, SC-560, PD98059) prior to dilution to the required concentration in cell culture media. On the day of the experiment 5 × 10^3^ differentiated PKiSS expressing SH-SY5Y cells/well in 96-well plates were pretreated with either media alone (control) or test drugs for a 2 h period. The fibrillar A*β* 1–40 (10 *μ*M) was then added to induce toxicity and cells were incubated for a further 16 hours prior to determination of cell viability. None of the solvents used (PBS, ddH2O, methanol, ethanol, or DMSO) had a statistically significant effect on cell viability or A*β* 1–40 (10 *μ*M) toxicity at a 1 : 100 dilution in cell culture medium.

### 2.8. Cell Viability

After treatment with test peptides or drugs and incubation for the appropriate time the viability was determined by either trypan blue dye exclusion with at least 100 cells counted per well or by 3-[4,5-dimethylthiazol-2-yl]- 2,5-diphenyltetrazolium bromide (MTT) reduction [24]. For MTT reduction determination, after incubation with test substances MTT (10 *μ*L: 12 mM stock) was added and cells incubated for a further 4 hours. Cell lysis buffer [100 *μ*L/well; 20% (v/v) SDS, 50% (v/v) N,N-dimethylformamide, pH 4.7] was added and after repeated pipetting to lyse cells the MTT formazan product formation was determined by measurement of absorbance change at 570 nm. Control levels in the absence of test substances were taken as 100% and the absorbance in the presence of cells lysed with Triton X-100 at the start of the incubation period with test substances taken as 0% [25]. All of the drugs tested had no statistically significant effect on the MTT assay in the absence or presence of cells. None of the solvents used (PBS, ddH2O, methanol, ethanol, or DMSO) had a statistically significant effect on the MTT assay in the absence or presence of cells.

### 2.9. Data Analysis

All data are expressed as means ± s.e.m. For cytotoxicity experiments data are expressed as % viable cells (trypan blue dye exclusion) or % control cells (MTT reduction). Statistical analysis was performed by one-way analysis of variance (ANOVA) due to the multiple variables (A*β*, test drug, and A*β* plus test drug being compared) using GraphPad Prism software (version 6). *Post hoc* analysis was carried with Tukey (for analysis of differences between KiSS-1 overexpressing and vector cells response to A*β*) or Dunnett (for comparisons involving test drugs) multiple comparison based on the recommendations of GraphPad Prism software for the data sets concerned, with a *P* value of <0.05 considered statistically significant.

## 3. Results

### 3.1. KiSS-1 Overexpression Cell Line Characterization

**Figure 1:**
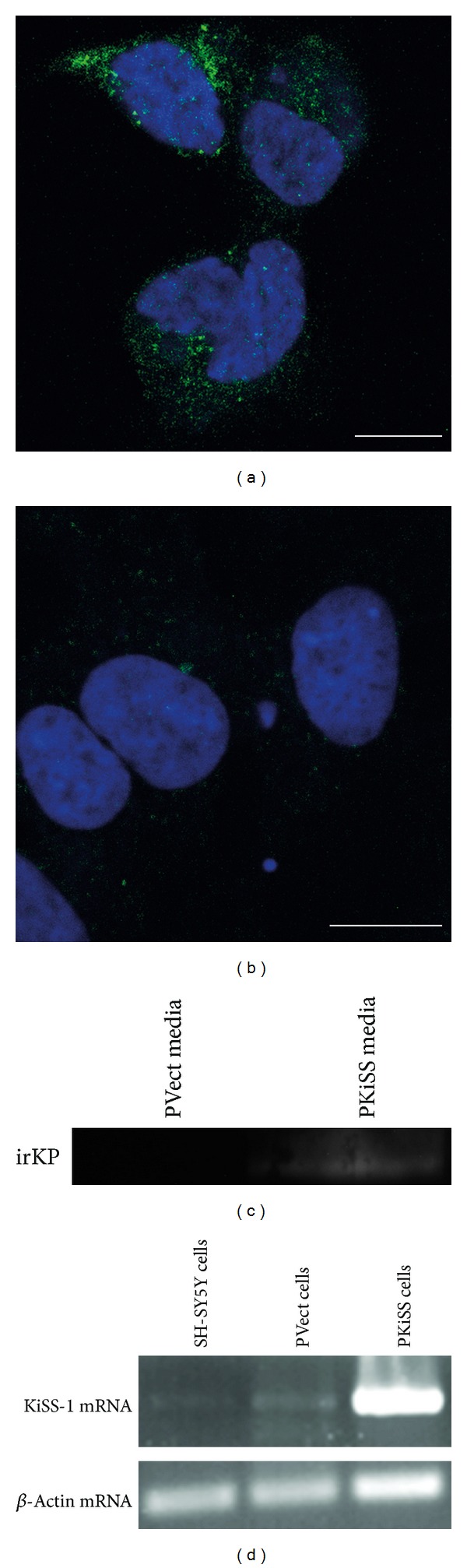
Characterization of KiSS-1 gene overexpression in SH-SY5Y neurons. (a) Immunocytochemistry of human SH-SY5Y neuron stable cell line containing the KiSS-1 gene vector (PKiSS) showing localization of kisspeptin in the cytoplasm. (b) Immunocytochemistry of human SH-SY5Y neuron stable cell line containing the pcDNA4/TO/myc-His expression vector (PVect) showing no localization of kisspeptin above background. KP appears green (anti-KP 45–54 staining) and the nucleus appears blue (TO-PRO-3 Iodide staining). Bars = 10 *μ*m. (c) Western blot of conditioned media from PKiSS and PVect cells showing ir-KP staining of a <10 kDa band in PKiSS media. (d) RT-PCR analysis of KiSS-1 and *β*-actin mRNA in human SH-SY5Y neurons, human SH-SY5Y neuron stable cell line containing the pcDNA4/TO/myc-His expression vector (PVect), and human SH-SY5Y neuron stable cell line containing the KiSS-1 gene vector (PKiSS).

The overexpression of the human KiSS-1 gene in the PKiSS SH-SY5Y neurons, stably transfected with the pcDNA4/TO/myc-His expression vector containing the human KiSS-1 gene, was confirmed using immunocytochemistry (Figure 1(a)), which showed that the anti-KP 45–54 staining was found within the cytoplasm. The staining of PVect control cells, stably transfected with the pcDNA4/TO/myc-His expression vector, showed no anti-KP 45–54 staining above the background levels (Figure 1(b)). Conditioned media from PKiSS SH-SY5Y neurons and PVect control cells were collected and the presence of immunoreactive (ir) KP was determined by western blotting. Results showed the presence of an ir-KP low molecular weight band (<10 kDa) in media from PKiSS SH-SY5Y neurons, that was not found in PVect control cells (Figure 1(c)). To confirm that the transfected KiSS-1 gene was expressed cells were analyzed by RT-PCR. Results showed a high level of KiSS-1 mRNA in the PKiSS SH-SY5Y neurons compared to that found in naive (untransfected) SH-SY5Y neurons and PVect SH-SY5Y neurons (Figure 1(d)).

### 3.2. Neuroprotection against Amyloid-*β* by KiSS-1 Overexpression and the Role of Kisspeptin

**Figure 2:**
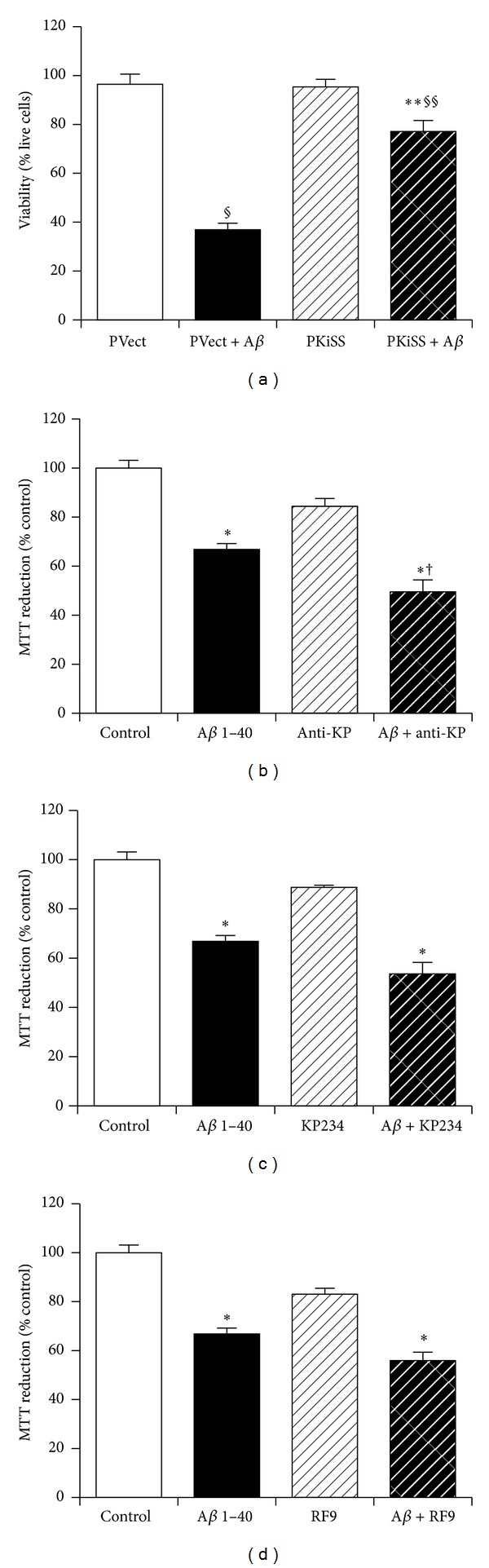
Effect of KiSS-1 gene overexpression on amyloid-*β* toxicity. (a) Human SH-SY5Y neurons stable cell lines containing control vector (PVect) or the KiSS-1 gene vector (PKiSS) were exposed to fibrillar A*β* 25–35 (10 *μ*M) and cell viability determined by trypan blue exclusion. PKiSS cells were pretreated with (b) anti-kisspeptin antibody (Anti-KP: 10 *μ*g/mL) or (c) kisspeptin receptor antagonist (KP234: 10 *μ*M) or (d) neuropeptide FF receptor antagonist (RF9: 10 *μ*M) for 2 h prior to exposure to fibrillar A*β* 1–40 (10 *μ*M) and determination of viability by MTT reduction. Results are mean ± s.e.m. (a) ^§^ *P* < 0.05 versus PVect; **P < 0.05 versus PKiSS; ^§§^ *P* < 0.05 versus PVect + A*β*; (b–d) *P < 0.05 versus control (media alone); ^†^ *P* < 0.05 versus A*β* alone; one-way ANOVA.

The overexpression of the KiSS-1 gene in SH-SY5Y neurons was shown to be significantly (*P* < 0.0001; one-way ANOVA, Tukey post hoc test) neuroprotective against A*β* 25–35 toxicity (Figure 2(a)), in agreement with the previous studies [1]. Pretreatment with anti-KP (10 *μ*g/mL), KP234 (10 *μ*M), and RF9 (10 *μ*M) was tested to confirm the observations from a previous study [1]. The anti-KP antibody significantly (*P* = 0.0421; one-way ANOVA, Dunnett post hoc test) enhanced the toxicity of A*β* 1–40 (10 *μ*M) in KiSS-1 overexpressing neurons (Figure 2(b)), whilst neither the KP receptor antagonist KP234 [26] (Figure 2(c)) nor the NPFF receptor antagonist RF9 [27] (Figure 2(d)) had any significant effect, in agreement with previous studies [1]. The anti-KP antibody, KP234, and RF9 had no effect on the KiSS-1 overexpressing neurons alone. The doses of anti-KP antibody, KP234, and RF9 were selected based on previous studies [1]. These results suggest that KP is the neuroprotective component derived from the KiSS-1 gene, confirming previous studies [1], and indicate that the neuroprotective actions of KP are not mediated via actions on either the KP or NPFF receptors. The results further suggest that another mechanism, possibly via a different receptor or protein interactions between KP and A*β*, may play a role.

### 3.3. The Role of Amyloid-Binding Interactions in Neuroprotection against Amyloid-*β* by KiSS-1 Overexpression

**Figure 3:**
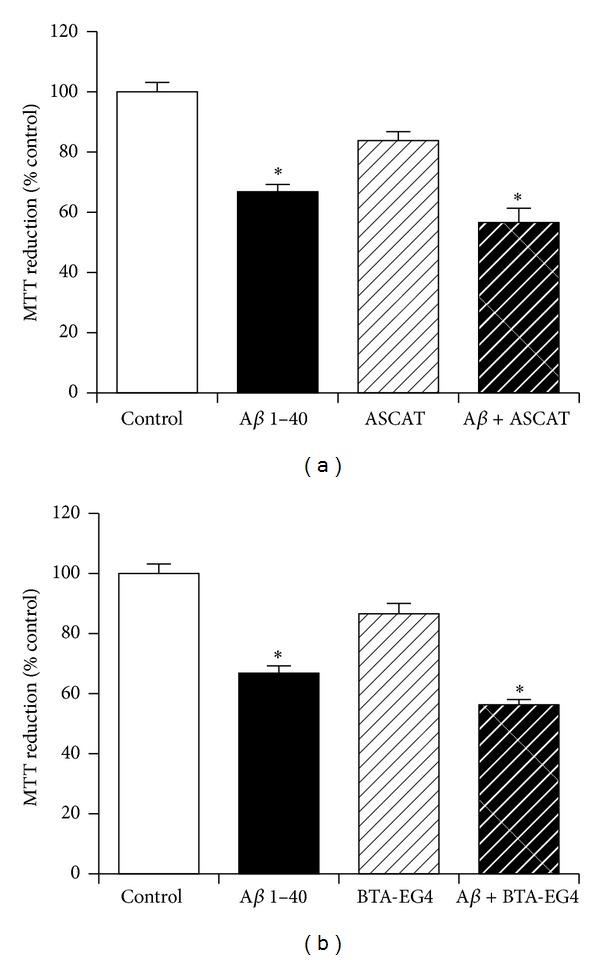
Effect of amyloid-binding compounds on KiSS-1 gene overexpression neuroprotection against amyloid-*β* toxicity. PKiSS cells were pretreated with (a) ASCAT peptide (100 *μ*M) or (b) BTA-EG4 (10 *μ*M) for 2 h prior to exposure to fibrillar A*β* 1–40 (10 *μ*M) and determination of viability by MTT reduction. Results are mean ± s.e.m. *P < 0.05 versus control (media alone); one-way ANOVA.

The KP peptides that are suggested to be the neuroprotective derivatives from KiSS-1 overexpression have been shown to specifically bind A*β*. A synthetic peptide, ASCAT, which contains an A*β*-like sequence [24] and competes with A*β* binding to KP was therefore tested. The dose chosen (100 *μ*M) has previously been shown to prevent A*β* inhibition of catalase without having a neuroprotective effect [24] and to prevent KP binding to amyloid peptides. Results showed that this compound had no significant effect on KiSS-1 overexpression induced neuroprotection against A*β* (Figure 3(a)). The BTA-EG4 compound, that has been developed as an A*β* binding agent [28], has previously been shown to displace A*β* binding to catalase [29] by binding the CA*β*BD of A*β* [24]. The dose chosen (10 *μ*M) prevents A*β* interactions with catalase but is not neuroprotective itself [29]. The KP peptide has been shown to bind the CA*β*BD of A*β* [1] and thus BTA-EG4 may displace KP binding to A*β*. When BTA-EG4 was tested in KiSS-1 overexpressing cells the compound had no effect of A*β* toxicity (Figure 3(b)). These results suggest that the mechanism for KiSS-1 neuroprotection against A*β* may not involve direct protein interactions between KP and A*β*.

### 3.4. The Role of Opioid Receptor Activation in Neuroprotection against Amyloid-*β* by KiSS-1 Overexpression

**Figure 4:**
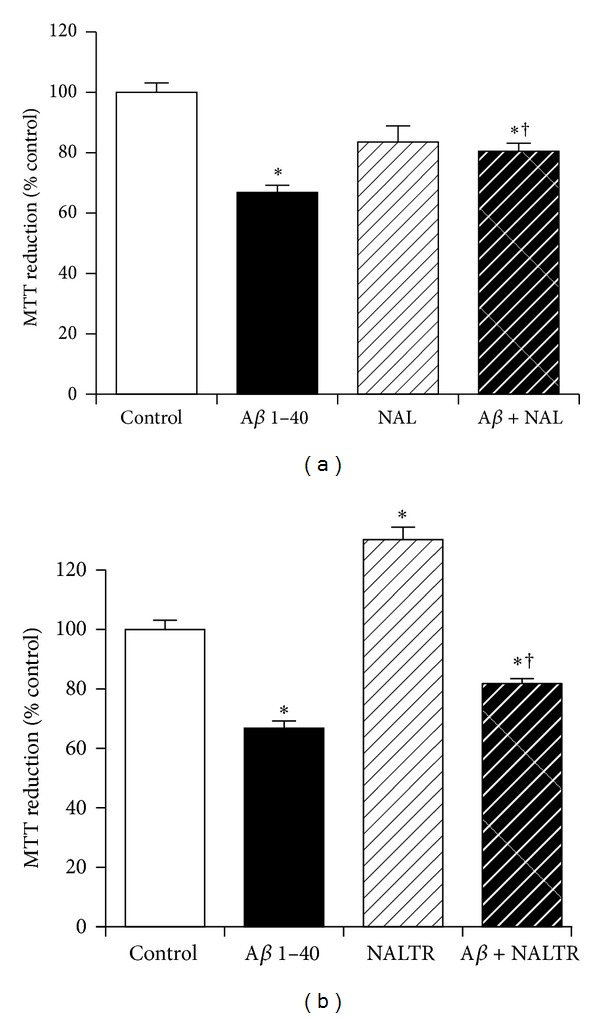
Effect of opioid receptor antagonism on KiSS-1 gene overexpression neuroprotection against amyloid-*β* toxicity. PKiSS cells were pretreated with (a) opioid antagonist naloxone (NAL: 1 *μ*M) or (b) opioid antagonist naltrexone (NALTR: 1 *μ*M) for 2 h prior to exposure to A*β* 1–40 (10 *μ*M) and determination of viability by MTT reduction. Results are mean ± s.e.m. *P < 0.05 versus control (media alone); ^†^ *P* < 0.05 versus A*β* alone; one-way ANOVA.

Opioids are neuroprotective against A*β* [30, 31] and also involved in KP activation of GnRH [12, 13]. The effects of the opioid receptor antagonists naloxone and naltrexone on KiSS-1 overexpression neuroprotection against A*β* were therefore tested. The doses of naloxone (1 *μ*M) and naltrexone (1 *μ*M) have previously been demonstrated to be effective in blocking the actions of opioids in cell culture models [30, 31]. Results showed that naloxone significantly (*P* = 0.0230; one-way ANOVA, Dunnett post hoc test) enhanced KiSS-1 overexpression neuroprotection against A*β* (Figure 4(a)). The naltrexone significantly (*P* < 0.0001; one-way ANOVA, Dunnett post hoc test) enhanced MTT reduction in control cells, suggesting that the compound had a proliferative effect on the KiSS-1 overexpressing neurons (Figure 4(b)). Naltrexone also significantly (*P* = 0.0086; one-way ANOVA, Dunnett post hoc test) enhanced KiSS-1 overexpression neuroprotection against A*β* (Figure 4(b)). 

### 3.5. The Role of Oxytocin in Neuroprotection against Amyloid-*β* by KiSS-1 Overexpression

**Figure 5:**
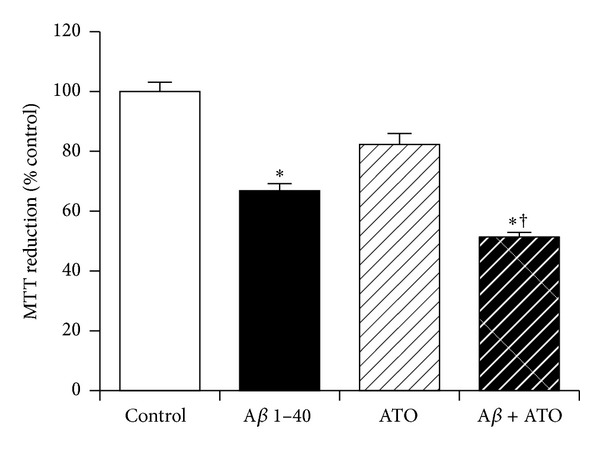
Effect of oxytocin receptor antagonism on KiSS-1 gene overexpression neuroprotection against amyloid-*β* toxicity. PKiSS cells were pretreated with atosiban (ATO: 1 *μ*M) for 2 h prior to exposure to A*β* 1–40 (10 *μ*M) and determination of viability by MTT reduction. Results are mean ± s.e.m. *P < 0.05 versus control (media alone); ^†^ *P* < 0.05 versus A*β* alone; one-way ANOVA.

The KP peptide is known to activate oxytocin *in vivo* [4]. The SH-SY5Y neurons express oxytocin receptors [32] and oxytocin has neuroprotective actions in this cell line [33]. The effects of atosiban, an antagonist of oxytocin [34], on KiSS-1 overexpression neuroprotection against A*β* were therefore tested at a dose (1 *μ*M) that is known to be effective in cell culture models. Results showed atosiban significantly (*P* = 0.0059; one-way ANOVA, Dunnett post hoc test) enhanced the toxicity of A*β* in KiSS-1 overexpressing neurons (Figure 5). This suggests that the oxytocin receptor system may play a role in KiSS-1 mediated neuroprotection.

### 3.6. The Role of Adrenergic Receptor Activation in Neuroprotection against Amyloid-*β* by KiSS-1 Overexpression

**Figure 6:**
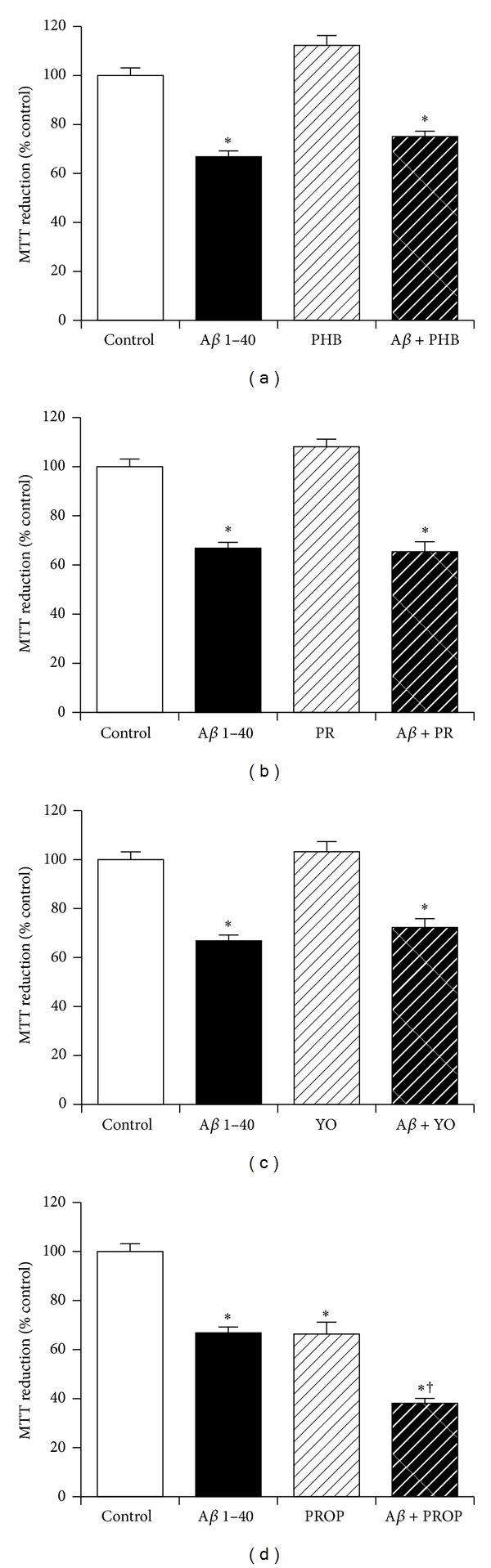
Effect of adrenergic receptor antagonism on KiSS-1 gene overexpression neuroprotection against amyloid-*β* toxicity. PKiSS cells were pretreated with (a) *α*-adrenergic antagonist phenoxybenzamine hydrochloride (PHB: 10 *μ*M), (b) *α*-adrenergic antagonist prazosin hydrochloride (PR: 250 nM), (c) *α*-adrenergic antagonist yohimbine hydrochloride (YO: 50 nM), (d) *β*-adrenergic antagonist propranolol hydrochloride (PROP: 50 nM) for 2 h prior to exposure to A*β* 1–40 (10 *μ*M) and determination of viability by MTT reduction. Results are mean ± s.e.m. *P < 0.05 versus control (media alone); ^†^ *P* < 0.05 versus A*β* alone; one-way ANOVA.

The KP peptide facilitates passive avoidance learning and memory consolidation *in vivo*, which can be inhibited by both *α*- and *β*-adrenergic antagonists [17]. The KP peptide also has antidepressant-like activity that can be inhibited by *α*2-adrenergic antagonists [16]. The effects of *α*- and *β*-adrenergic antagonists on KiSS-1 overexpression neuroprotection against A*β* were therefore tested. The doses of phenoxybenzamine hydrochloride (10 *μ*M), prazosin hydrochloride (250 nM), yohimbine hydrochloride (50 nM), and propranolol hydrochloride (50 nM) have previously been demonstrated to be effective in neuronal cell culture models. Results showed that the *α*-adrenergic antagonists phenoxybenzamine hydrochloride (Figure 6(a)), prazosin hydrochloride (Figure 6(b)), and yohimbine hydrochloride (Figure 6(c)) had no significant effect on the toxicity of A*β* in KiSS-1 overexpressing neurons. The *β*-adrenergic antagonist propranolol hydrochloride caused a significant (*P* < 0.0001; one-way ANOVA, Dunnett post hoc test) reduction in the viability of KiSS-1 overexpressing SH-SY5Y neurons (Figure 6(d)), at a dose that is nontoxic to SH-SY5Y neurons [35]. The propranolol also caused a significant (*P* < 0.0001; one-way ANOVA, Dunnett post hoc test) enhancement of A*β* toxicity in the KiSS-1 overexpressing neurons (Figure 6(d)), suggesting that the toxicity of propranolol in the KiSS-1 overexpressing cells was additive to the toxicity of A*β*.

### 3.7. The Role of Cholinergic Receptor Activation in Neuroprotection against Amyloid-*β* by KiSS-1 Overexpression

**Figure 7:**
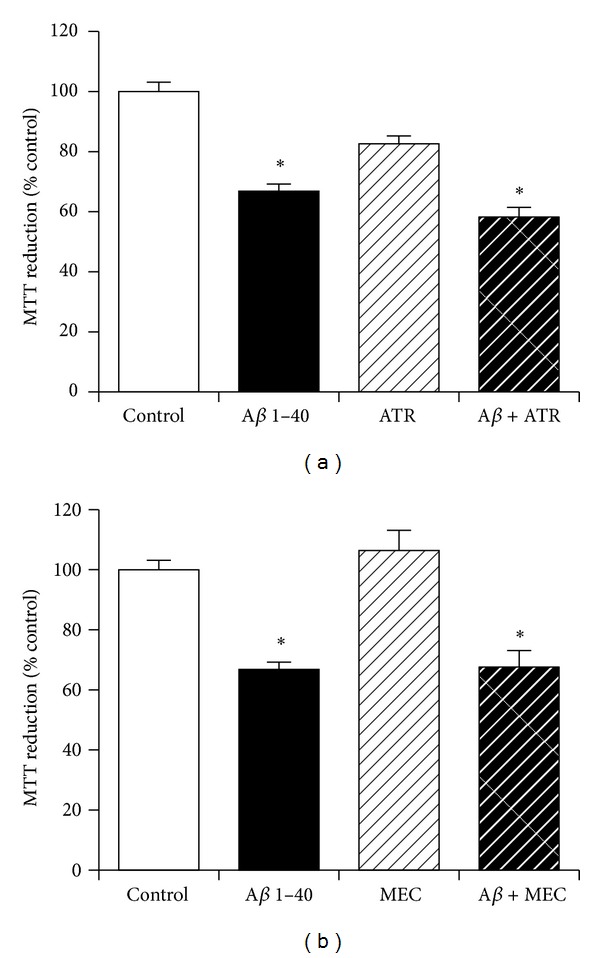
Effect of cholinergic receptor antagonism on KiSS-1 gene overexpression neuroprotection against amyloid-*β* toxicity. PKiSS cells were pretreated with (a) muscarinic acetylcholine antagonist atropine sulphate (ATR: 10 *μ*M) or (b) nicotinic acetylcholine antagonist mecamylamine hydrochloride (MEC: 10 *μ*M) for 2 h prior to exposure to A*β* 1–40 (10 *μ*M) and determination of viability by MTT reduction. Results are mean ± s.e.m. *P < 0.05 versus control (media alone); one-way ANOVA.

The KP peptide facilitates passive avoidance learning and memory consolidation *in vivo*, which can be inhibited by muscarinic but not nicotinic cholinergic antagonists [17]. The effects of muscarinic and nicotinic cholinergic antagonists on KiSS-1 overexpression neuroprotection against A*β* were therefore tested. The doses of atropine sulphate (10 *μ*M) and mecamylamine hydrochloride (10 *μ*M) have previously been demonstrated to be effective in neuronal cell culture models. Results showed that the muscarinic acetylcholine antagonist atropine sulphate (Figure 7(a)) and the nicotinic acetylcholine antagonist mecamylamine hydrochloride (Figure 7(b)) had no significant effect on the toxicity of A*β* in KiSS-1 overexpressing neurons.

### 3.8. The Role of Dopaminergic Receptor Activation in Neuroprotection against Amyloid-*β* by KiSS-1 Overexpression

**Figure 8:**
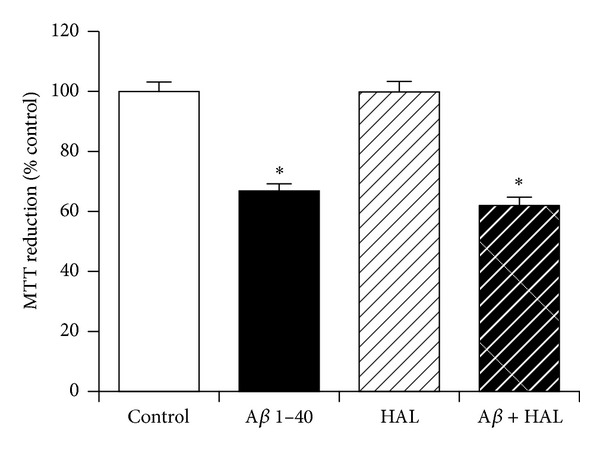
Effect of dopaminergic receptor antagonism on KiSS-1 gene overexpression neuroprotection against amyloid-*β* toxicity. PKiSS cells were pretreated with haloperidol (HAL: 10 *μ*M) for 2 h prior to exposure to A*β* 1–40 (10 *μ*M) and determination of viability by MTT reduction. Results are mean ± s.e.m. *P < 0.05 versus control (media alone); one-way ANOVA.

The KP system is known to modulate dopamine levels [36] and some neurons coexpress KP plus dopamine synthesis enzymes [37]. The SH-SY5Y neuroblastoma is dopaminergic [38] and the effect of the dopaminergic antagonist haloperidol was therefore tested on KiSS-1 overexpression neuroprotection against A*β*. The dose of haloperidol (10 *μ*M) has previously been demonstrated to be effective in neuronal cell culture models. Results showed that haloperidol had no significant effect on the toxicity of A*β* in KiSS-1 overexpressing neurons (Figure 8). The dopaminergic antagonist haloperidol has also been suggested to have neuroprotective actions against A*β* [39], an effect not observed in the KiSS-1 overexpressing neurons.

### 3.9. The Role of Serotonergic Receptor Activation in Neuroprotection against Amyloid-*β* by KiSS-1 Overexpression

**Figure 9:**
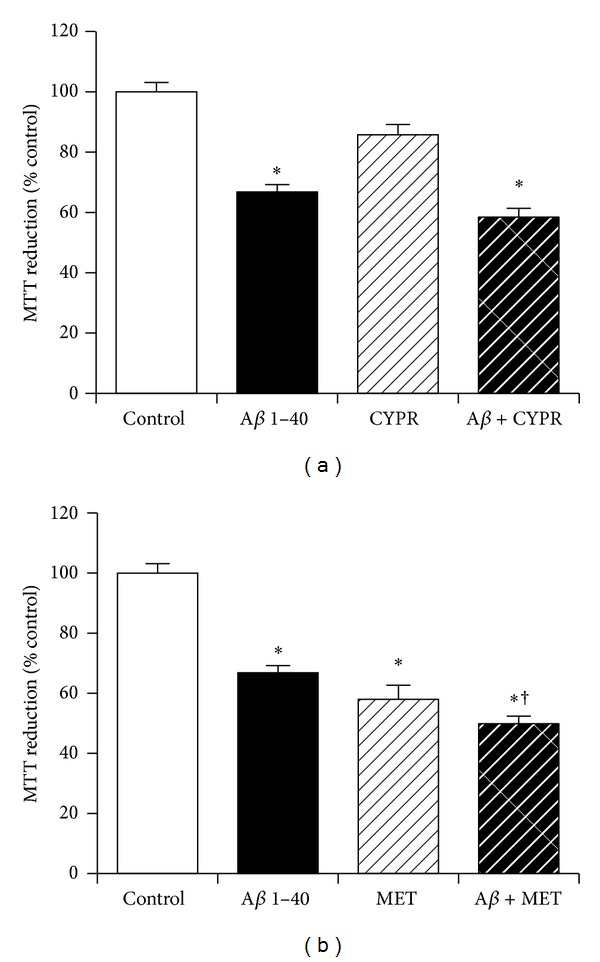
Effect of serotonergic receptor antagonism on KiSS-1 gene overexpression neuroprotection against amyloid-*β* toxicity. PKiSS cells were pretreated with (a) serotonergic antagonist cyproheptadine hydrochloride (CYPR: 10 nM) or (b) serotonergic antagonist methysergide maleate (MET: 1 *μ*M) for 2 h prior to exposure to A*β* 1–40 (10 *μ*M) and determination of viability by MTT reduction. Results are mean ± s.e.m. *P < 0.05 versus control (media alone); ^†^ *P* < 0.05 versus A*β* alone; one-way ANOVA.

The KP peptide facilitates passive avoidance learning and memory consolidation *in vivo*, which can be inhibited by 5-HT2 serotonergic antagonists [17]. The KP peptide also has antidepressant-like activity that can be inhibited by 5-HT2 serotonergic receptor antagonists [16]. The effects of serotonergic receptor antagonists on KiSS-1 overexpression neuroprotection against A*β* were therefore tested. The doses of cyproheptadine hydrochloride (10 nM) and methysergide maleate (1 *μ*M) have previously been demonstrated to be effective in neuronal cell culture models. Results showed the 5-HT2 serotonergic antagonist cyproheptadine hydrochloride (Figure 9(a)) had no significant effect on the toxicity of A*β* in KiSS-1 overexpressing neurons. The mixed 5-HT1/5-HT2 receptor antagonist methysergide maleate caused a significant (*P* < 0.0001; one-way ANOVA, Dunnett post hoc test) reduction in the viability of KiSS-1 overexpressing SH-SY5Y neurons (Figure 9(b)), at a dose that is nontoxic to neuronal cell lines [40]. The methysergide maleate also caused a significant (*P* = 0.0016; one-way ANOVA, Dunnett post hoc test) enhancement of A*β* toxicity in the KiSS-1 overexpressing neurons (Figure 9(b)), suggesting that the toxicity of methysergide maleate in the KiSS-1 overexpressing cells was additive to the toxicity of A*β*.

### 3.10. The Role of GABA-A Receptor Activation in Neuroprotection against Amyloid-*β* by KiSS-1 Overexpression

**Figure 10:**
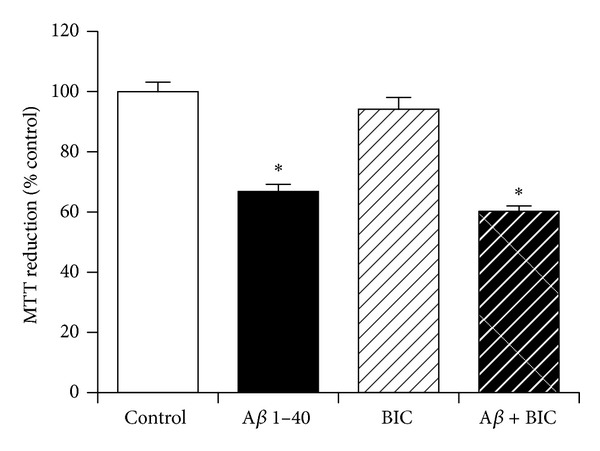
Effect of GABA receptor antagonism on KiSS-1 gene overexpression neuroprotection against amyloid-*β* toxicity. PKiSS cells were pretreated with the GABA-A antagonist 1(S),9(R)-(−)-bicuculline methiodide (BIC: 50 *μ*M) for 2 h prior to exposure to A*β* 1–40 (10 *μ*M) and determination of viability by MTT reduction. Results are mean ± s.e.m. *P < 0.05 versus control (media alone); one-way ANOVA.

The KP peptide facilitates passive avoidance learning and memory consolidation *in vivo*, which can be inhibited by the GABA-A antagonist bicuculline [17]. The effect of bicuculline on KiSS-1 overexpression neuroprotection against A*β* was therefore tested. The dose of 1(S),9(R)-(−)-bicuculline methiodide (50 *μ*M) has previously been demonstrated to be effective in neuronal cell culture models. Results showed the bicuculline had no significant effect on the toxicity of A*β* in KiSS-1 overexpressing neurons (Figure 10).

### 3.11. The Role of Estrogen Receptor Activation in Neuroprotection against Amyloid-*β* by KiSS-1 Overexpression

**Figure 11:**
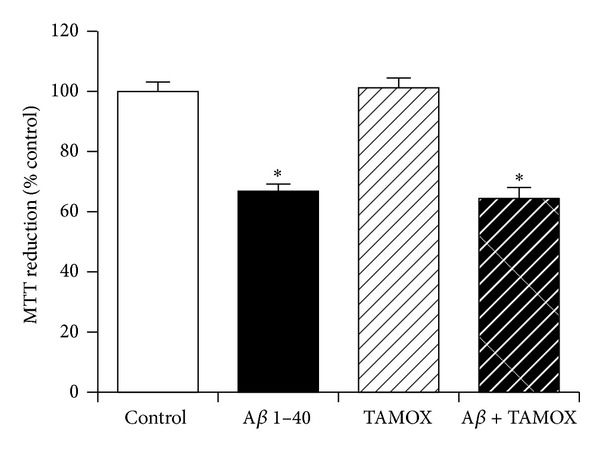
Effect of estrogen receptor antagonism on KiSS-1 gene overexpression neuroprotection against amyloid-*β* toxicity. PKiSS cells were pretreated with the estrogen antagonist tamoxifen (TAMOX: 10 *μ*M) for 2 h prior to exposure to A*β* 1–40 (10 *μ*M) and determination of viability by MTT reduction. Results are mean ± s.e.m. *P < 0.05 versus control (media alone); one-way ANOVA.

Activation of estrogen receptors is known to alter KP levels [41, 42] and also plays a role in the neuroprotection against A*β* [31, 43]. The effect of the estrogen receptor antagonist tamoxifen on KiSS-1 overexpression neuroprotection against A*β* was therefore tested. The dose of tamoxifen (10 *μ*M) has previously been demonstrated to be effective in neuronal cell culture models. Results showed that tamoxifen had no significant effect on the toxicity of A*β* in KiSS-1 overexpressing neurons (Figure 11).

### 3.12. The Role of Catalase, Cyclooxygenase, Nitric Oxide Synthase, and *γ*-Secretase Enzymes in Neuroprotection against Amyloid-*β* by KiSS-1 Overexpression

**Figure 12:**
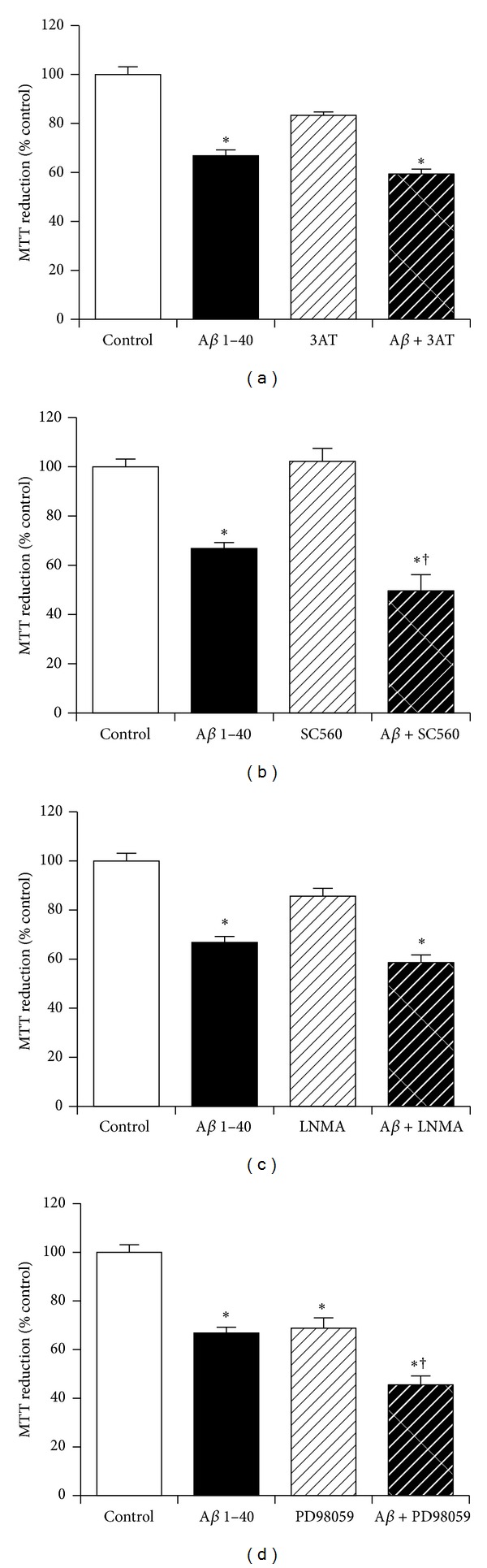
Effect of endogenous enzyme inhibition on KiSS-1 gene overexpression neuroprotection against amyloid-*β* toxicity. PKiSS cells were pretreated with (a) catalase inhibitor 3-Amino-1,2,4-triazole (3AT: 50 mM), (b) cyclooxygenase inhibitor SC560 (1 *μ*M), (c) nitric oxide synthase inhibitor N^G^-Methyl-L-arginine acetate salt (LNMA: 1 mM), (d) mitogen activated protein kinase cascade inhibitor PD98059 (50 *μ*M) for 2 h prior to exposure to A*β* 1–40 (10 *μ*M) and determination of viability by MTT reduction. Results are mean ± s.e.m. *P < 0.05 versus control (media alone); ^†^ *P* < 0.05 versus A*β* alone; one-way ANOVA.

The KP peptide is known to increase catalase activity [18], which is also neuroprotective against A*β* [44]. The KP peptide also has thermoregulatory effects [19] and acts via nitric oxide in the facilitation of passive avoidance learning plus memory consolidation *in vivo* [17]. Another possible mechanism for the neuroprotective action of KiSS-1 overexpression is via activation of intracellular second messenger pathways. The effects of catalase inhibition, cyclooxygenase inhibition, nitric oxide synthase inhibition, and also the mitogen activated protein kinase cascade inhibitor PD98059 on KiSS-1 overexpression neuroprotection against A*β* were tested to determine if these processes were involved. The doses of 3-Amino-1,2,4-triazole (50 mM), SC-560 (1 *μ*M), N^G^-Methyl-L-arginine acetate salt (1 mM), and PD98059 (50 *μ*M) have previously been demonstrated to be effective in neuronal cell culture models. Results showed that catalase inhibition with 3-Amino-1,2,4-triazole had no effect on KiSS-1 overexpression neuroprotection against A*β* (Figure 12(a)). The cyclooxygenase-1 inhibitor SC-560 significantly (*P* = 0.0029; one-way ANOVA, Dunnett post hoc test) reduced KiSS-1 overexpression neuroprotection against A*β* (Figure 12(b)). Nitric oxide synthase inhibition with N^G^-Methyl-L-arginine acetate had no effect on KiSS-1 overexpression neuroprotection against A*β* (Figure 12(c)). The mitogen activated protein kinase cascade inhibitor PD98059 caused a significant (*P* < 0.0001; one-way ANOVA, Dunnett post hoc test) reduction in the viability of KiSS-1 overexpressing SH-SY5Y neurons (Figure 12(d)), at a dose that has no effect on SH-SY5Y neurons [45]. The PD98059 also caused a significant (*P* < 0.0001; one-way ANOVA, Dunnett post hoc test) enhancement of A*β* toxicity in the KiSS-1 overexpressing neurons (Figure 12(d)), suggesting that the toxicity of PD98059 in the KiSS-1 overexpressing cells was additive to the toxicity of A*β* rather than KiSS-1 neuroprotection being mediated via activation of the mitogen activated protein kinase cascade.

## 4. Discussion

The effects of the anti-KP antibody on KiSS-1 overexpression neuroprotection against A*β* have previously been reported and the mechanism of neuroprotection by KP has been suggested not to involve either the KP or NPFF receptors [1]. The failure of ASCAT and BTA-EG4 compounds, that modify KP binding to A*β*, to modulate this process suggests that the proposed binding interaction may not mediate the neuroprotection in this system. The levels of KP released by SH-SY5Y neurons in response to A*β* are likely to be insufficient to provide full neuroprotection via a binding action [1, 11]. However, in the KiSS-1 overexpressing neurons there is a significant release of an ir-KP-like material into the media that could either bind A*β* or activate receptor mediated pathways. It is therefore likely that the mechanism for neuroprotection may involve an alternative process that is more likely receptor mediated. The *in vivo* actions of KP peptides include actions on the opioid system [12, 13], oxytocin/vasopressin systems [4, 14, 15], neurotransmitter systems [16, 17], activation of endogenous antioxidants [18], activation of nitric oxide [17], and effects on thermoregulation [19] that could be mediated via the prostaglandin systems [46, 47].

The naloxone and naltrexone reduction in the toxicity of A*β* raises the possibility that endogenous opioids may play a role in the toxicity of A*β*. Similar effects were observed with naloxone and naltrexone on A*β* toxicity; however, these opioid antagonists had different effects on cell viability itself which complicated the interpretation of the results. The antiopioid activity of KP peptides has been suggested by their activation of NPFF receptors [8, 9] and the KiSS-1 derivative KSO also acts as an NPFF ligand [10]. However, the NPFF antagonist RF9 had no effect on KiSS-1 overexpression neuroprotection against A*β*. The RF9 is known to block the antiopioid activity of NPFF [48] but has recently been suggested to be ineffective at blocking all the actions of NPFF and related peptides [49]. As such the effects of KiSS-1 overexpression on A*β* toxicity are unlikely to involve a partial suppression of endogenous opioid actions by KP that is enhanced by naloxone or naltrexone.

The effects of atosiban suggest a role for the oxytocin system in the neuroprotection provided by KiSS-1 overexpression. The actions of atosiban also include inhibition of vasopressin receptors [50] and it is known that some of the actions of KP peptides are mediated via actions on vasopressin [14]. *In vivo* KP activates both oxytocin and vasopressin [4, 14, 15], as such it is possible that either or both the oxytocin and vasopressin systems are involved in KiSS-1 neuroprotection. 

From this study the adrenergic, cholinergic, dopaminergic, serotonergic, and GABA neurotransmitter systems plus the nitric oxide and estrogen receptor activated systems do not appear to play a role in the neuroprotective actions of KiSS-1 overexpression against the A*β* peptide. The *β*-adrenergic antagonist propranolol hydrochloride and the mixed 5-HT1/5-HT2 receptor antagonist methysergide maleate both had toxic actions in KiSS-1 overexpressing neurons at concentrations that are not toxic to SH-SY5Y neurons [35, 40]. The *β*-adrenergic antagonist propranolol hydrochloride and the mixed 5-HT1/5-HT2 receptor antagonist methysergide maleate also enhanced A*β* toxicity; however, this is more likely due to the toxicity of these antagonists to KiSS-1 overexpressing neurons rather than the involvement of noradrenaline or serotonin in the KiSS-1 mediated neuroprotection. Both noradrenaline [51, 52] and serotonin [53] have neuroprotective properties. The mitogen activated protein kinase cascade inhibitor PD98059 also inhibited cell viability and the *β*-adrenergic [54] plus 5HT1 serotonergic [55] receptors can act via the mitogen activated protein kinase cascade. Since the KP peptide is known to activate both *β*-adrenergic and serotonergic pathways *in vivo* [17] it is possible that these pathways are upregulated in this overexpression system and play a role in the neuronal survival. The mitogen activated protein kinase cascade may provide the second messenger system for the *β*-adrenergic plus 5HT1 serotonergic pathways involved.

The mitogen activated protein kinase cascade inhibitor PD98059 has previously been shown to reduce the anti-A*β* effects of a number of neuroprotective compounds [56–60]. PD98059 also attenuates KP induced modulation of GnRH mRNA [61] and KP upregulation of excitatory synaptic transmission [62].

The SC-560 cyclooxygenase-1 inhibitor has previously been shown to reduce A*β* production in an AD model [63]. The specificity of SC-560 for cyclooxygenase-1 over cyclooxygenase-2 is altered in some cell systems [64] and it is unknown which form of cyclooxygenase contributes in the KiSS-1 overexpression model. The ability of this compound to enhance A*β* toxicity in the KiSS-1 overexpression model suggests that there may be modulation of the cyclooxygenase system in these neurons. The ability of KP to modulate thermoregulatory responses *in vivo* [19] could be modulated via cyclooxygenase inhibitors [46, 47]. As such KP could be acting via prostaglandin synthesis in this overexpression model and *in vivo*. 

**Figure 13:**
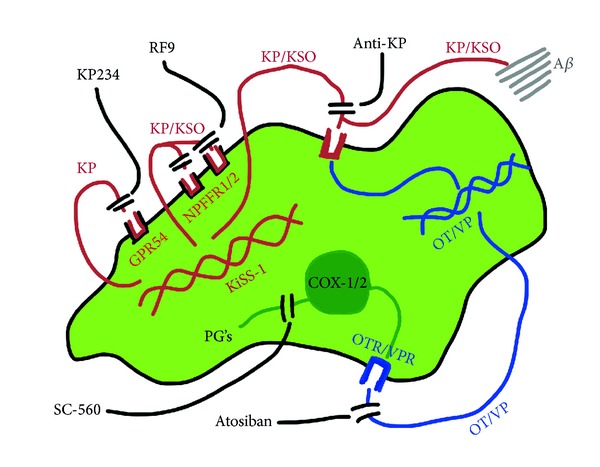
Model for KiSS-1 gene overexpression neuroprotective mechanisms against amyloid-*β* toxicity. KP: kisspeptin; KSO: kissorphin; KP234: kisspeptin receptor antagonist; GPR54: kisspeptin receptor; RF9: neuropeptide FF receptor antagonist; NPFFR1/2: neuropeptide FF receptors 1 and 2; Anti-KP: anti-kisspeptin 45–54 antiserum; A*β*: amyloid-*β*; OT: oxytocin; VP: vasopressin; atosiban: oxytocin/vasopressin receptor antagonist; OTR: oxytocin receptor; VPR: vasopressin receptor; COX-1/2: cyclooxygenase 1 and 2; PG's: prostaglandins; and SC-560: cyclooxygenase 1 antagonist.

The observation that KiSS-1 neuroprotection has both an oxytocin/vasopressin plus a cyclooxygenase dependent component could be due to endogenous oxytocin or vasopressin activating cyclooxygenase. Both *in vivo* adminstration and *in vitro* administration of oxytocin [65, 66] or vasopressin [67, 68] causes an activation of prostaglandin synthesis that is cyclooxygenase dependent. The SH-SY5Y neuronal cell line is known to express the vasopressin gene [69] suggesting that this could be a source of the endogenous material antagonized by atosiban. This proposed mechanism for KiSS-1 mediated neuroprotection against A*β* is summarized in Figure 13. The action of KP or KSO products of the KiSS-1 gene appears to be independent of the KP and NPFF receptors and a direct binding action on A*β* cannot be excluded [1] but at the concentrations of KP found in the system is unlikely to have a major effect.

## 5. Conclusion

KiSS-1 overexpression is neuroprotective against A*β* and the action appears to involve the KP peptide product of KiSS-1 processing, which is released by the cells. The mechanism of neuroprotection does not involve the KP or NPFF receptors. Opioids play a role in the toxicity of A*β* in the KiSS-1 overexpression system. The mechanism of protection appears to have an oxytocin/vasopressin plus a cyclooxygenase dependent component, which may be linked and can be blocked by the oxytocin/vasopressin antagonist atosiban or the cyclooxygenase-1 antagonist SC-560 (Figure 13). The contribution of KP binding to A*β* may also contribute to the neuroprotection observed in this model [1].

## References

[1] Milton NGN, Chilumuri A, Rocha-Ferreira E, Nercessian AN, Ashioti M (2012). Kisspeptin prevention of amyloid-*β* Peptide neurotoxicity *in vitro*. *ACS Chemical Neuroscience*.

[2] Navarro VM, Tena-Sempere M (2012). Neuroendocrine control by kisspeptins: role in metabolic regulation of fertility. *Nature Reviews Endocrinology*.

[3] Kirby HR, Maguire JJ, Colledge WH, Davenport AP (2010). International Union of Basic and Clinical Pharmacology. LXXVII. Kisspeptin receptor nomenclature, distribution, and function. *Pharmacological Reviews*.

[4] Kotani M, Detheux M, Vandenbogaerde A (2001). The metastasis suppressor gene KiSS-1 encodes kisspeptins, the natural ligands of the orphan G protein-coupled receptor GPR54. *Journal of Biological Chemistry*.

[5] Bilban M, Ghaffari-Tabrizi N, Hintermann E (2004). Kisspeptin-10, a KiSS-1/metastin-derived decapeptide, is a physiological invasion inhibitor of primary human trophoblasts. *Journal of Cell Science*.

[6] Muir AI, Chamberlain L, Elshourbagy NA (2001). AXOR12, a novel human G protein-coupled receptor, activated by the peptide KiSS-1. *Journal of Biological Chemistry*.

[7] Ohtaki T, Shintani Y, Honda S (2001). Metastasis suppressor gene KiSS-1 encodes peptide ligand of a G-protein-coupled receptor. *Nature*.

[8] Lyubimov Y, Engström M, Wurster S, Savola J-M, Korpi ER, Panula P (2010). Human kisspeptins activate neuropeptide FF2 receptor. *Neuroscience*.

[9] Oishi S, Misu R, Tomita K (2011). Activation of neuropeptide FF receptors by kisspeptin receptor ligands. *ACS Medicinal Chemistry Letters*.

[10] Milton NGN (2012). *In vitro* activities of kissorphin, a novel hexapeptide KiSS-1 derivative, in neuronal cells. *Journal of Amino Acids*.

[11] Chilumuri A, Ashioti M, Nercessian AN, Milton NGN (2013). Immunolocalization of kisspeptin associated with amyloid-*β* deposits in the pons of an Alzheimer's disease patient. *Journal of Neurodegenerative Diseases*.

[12] Lehman MN, Coolen LM, Goodman RL (2010). Minireview: kisspeptin/neurokinin B/dynorphin (KNDy) cells of the arcuate nucleus: a central node in the control of gonadotropin-releasing hormone secretion. *Endocrinology*.

[13] Mostari MP, Ieda N, Deura C (2013). Dynorphin-kappa opioid receptor signaling partly mediates estrogen negative feedback effect on LH pulses in female rats. *Journal of Reproduction and Development*.

[14] Han X-F, Yan M, An X-F, He M, Yu J-Y (2010). Central administration of kisspeptin-10 inhibits natriuresis and diuresis induced by blood volume expansion in anesthetized male rats. *Acta Pharmacologica Sinica*.

[15] Scott V, Brown CH (2011). Kisspeptin activation of supraoptic nucleus neurons *in vivo*. *Endocrinology*.

[16] Tanaka M, Csabafi K, Telegdy G (2013). Neurotransmissions of antidepressant-like effects of kisspeptin-13. *Regulatory Peptides*.

[17] Telegdy G, Adamik A (2013). The action of kisspeptin-13 on passive avoidance learning in mice. Involvement of transmitters. *Behavioural Brain Research*.

[18] Aydin M, Oktar S, Yonden Z, Ozturk OH, Yilmaz B (2010). Direct and indirect effects of kisspeptin on liver oxidant and antioxidant systems in young male rats. *Cell Biochemistry and Function*.

[19] Csabafi K, Jászberényi M, Bagosi Z, Lipták N, Telegdy G (2013). Effects of kisspeptin-13 on the hypothalamic-pituitary-adrenal axis, thermoregulation, anxiety and locomotor activity in rats. *Behavioural Brain Research*.

[20] Milton NGN, Harris JR (2009). Polymorphism of amyloid-*β* fibrils and its effects on human erythrocyte catalase binding. *Micron*.

[21] Milton NGN, Harris JR (2010). Human islet amyloid polypeptide fibril binding to catalase: a transmission electron microscopy and microplate study. *TheScientificWorldJOURNAL*.

[22] Milton NGN, Harris JR (2013). Fibril formation and toxicity of the non-amyloidogenic rat amylin peptide. *Micron*.

[23] Milton NGN (2008). Homocysteine inhibits hydrogen peroxide breakdown by catalase. *The Open Enzyme Inhibition Journal*.

[24] Milton NGN, Mayor NP, Rawlinson J (2001). Identification of amyloid-*β* binding sites using an antisense peptide approach. *NeuroReport*.

[25] Milton NGN, Harris JR, Graham JM, Rickwood D (2006). Amyloid-*β* phosphorylation. *Cell Biology Protocols*.

[26] Roseweir AK, Kauffman AS, Smith JT (2009). Discovery of potent kisspeptin antagonists delineate physiological mechanisms of gonadotropin regulation. *Journal of Neuroscience*.

[27] Simonin F, Schmitt M, Laulin J-P (2006). RF9, a potent and selective neuropeptide FF receptor antagonist, prevents opioid-induced tolerance associated with hyperalgesia. *Proceedings of the National Academy of Sciences of the United States of America*.

[28] Inbar P, Li CQ, Takayama SA, Bautista MR, Yang J (2006). Oligo(ethylene glycol) derivatives of thioflavin T as inhibitors of protein-amyloid interactions. *ChemBioChem*.

[29] Habib LK, Lee MTC, Yang J (2010). Inhibitors of catalase-amyloid interactions protect cells from *β*-amyloid-induced oxidative stress and toxicity. *Journal of Biological Chemistry*.

[30] Szegedi V, Juhász G, Rózsa E (2006). Endomorphin-2, an endogenous tetrapeptide, protects against Abeta1-42 *in vitro* and *in vivo*. *The FASEB Journal*.

[31] Cui J, Wang Y, Dong Q (2011). Morphine protects against intracellular amyloid toxicity by inducing estradiol release and upregulation of Hsp70. *Journal of Neuroscience*.

[32] Cassoni P, Sapino A, Stella A, Fortunati N, Bussolati G (1998). Presence and significance of oxytocin receptors in human neuroblastomas and glial tumors. *International Journal of Cancer*.

[33] Bakos J, Strbak V, Ratulovska N, Bacova Z (2012). Effect of oxytocin on neuroblastoma cell viability and growth. *Cellular and Molecular Neurobiology*.

[34] Bakos J, Strbak V, Paulikova H, Krajnakova L, Lestanova Z, Bacova Z (2013). Oxytocin receptor ligands induce changes in cytoskeleton in neuroblastoma cells. *Journal of Molecular Neuroscience*.

[35] Mikami M, Goubaeva F, Song JH, Lee HT, Yang J (2008). *β*-adrenoceptor blockers protect against staurosporine-induced apoptosis in SH-SY5Y neuroblastoma cells. *European Journal of Pharmacology*.

[36] Szawka RE, Ribeiro AB, Leite CM (2010). Kisspeptin regulates prolactin release through hypothalamic dopaminergic neurons. *Endocrinology*.

[37] Clarkson J, Herbison AE (2011). Dual phenotype kisspeptin-dopamine neurones of the rostral periventricular area of the third ventricle project to gonadotrophin-releasing hormone neurones. *Journal of Neuroendocrinology*.

[38] Xie H-R, Hu L-S, Li G-Y (2010). SH-SY5Y human neuroblastoma cell line: *in vitro* cell model of dopaminergic neurons in Parkinson’s disease. *Chinese Medical Journal*.

[39] Yang M-C, Lung F-W (2011). Neuroprotection of paliperidone on SH-SY5Y cells against *β*-amyloid peptide25-35, N-methyl-4-phenylpyridinium ion, and hydrogen peroxide-induced cell death. *Psychopharmacology*.

[40] Lambert JJ, Peters JA, Hales TG, Dempster J (1989). The properties of 5-HT3 receptors in clonal cell lines studied by patch-clamp techniques. *British Journal of Pharmacology*.

[41] Smith JT, Cunningham MJ, Rissman EF, Clifton DK, Steiner RA (2005). Regulation of Kiss1 gene expression in the brain of the female mouse. *Endocrinology*.

[42] Alçin E, Sahu A, Ramaswamy S (2013). Ovarian regulation of kisspeptin neurones in the arcuate nucleus of the rhesus monkey (*Macaca mulatta*). *Journal of Neuroendocrinology*.

[43] Zhang Y, Champagne N, Beitel LK, Goodyer CG, Trifiro M, LeBlanc A (2004). Estrogen and androgen protection of human neurons against intracellular amyloid *β*1-42 toxicity through heat shock protein 70. *Journal of Neuroscience*.

[44] Milton NGN (2001). Inhibition of catalase activity with 3-amino-triazole enhances the cytotoxicity of the Alzheimer’s amyloid-*β* peptide. *NeuroToxicology*.

[45] Tang L-L, Wang R, Tang X-C (2005). Huperzine A protects SHSY5Y neuroblastoma cells against oxidative stress damage via nerve growth factor production. *European Journal of Pharmacology*.

[46] Nakamura K (2011). Central circuitries for body temperature regulation and fever. *American Journal of Physiology*.

[47] Morrison SF, Madden CJ, Tupone D (2012). Central control of brown adipose tissue thermogenesis. *Frontiers in Endocrinology*.

[48] Han ZL, Wang ZL, Tang HZ (2013). Neuropeptide FF attenuates the acquisition and the expression of conditioned place aversion to endomorphin-2 in mice. *Behavioural Brain Research*.

[49] Maletínská L, Tichá A, Nagelová V (2013). Neuropeptide FF analog RF9 is not an antagonist of NPFF receptor and decreases food intake in mice after its central and peripheral administration. *Brain Research*.

[50] Manning M, Cheng LL, Stoev S (2005). Design of peptide oxytocin antagonists with strikingly higher affinities and selectivities for the human oxytocin receptor than atosiban. *Journal of Peptide Science*.

[51] Madrigal JLM, Kalinin S, Richardson JC, Feinstein DL (2007). Neuroprotective actions of noradrenaline: effects on glutathione synthesis and activation of peroxisome proliferator activated receptor delta. *Journal of Neurochemistry*.

[52] Madrigal JLM, Leza JC, Polak P, Kalinin S, Feinstein DL (2009). Astrocyte-derived MCP-1 mediates neuroprotective effects of noradrenaline. *Journal of Neuroscience*.

[53] Fricker AD, Rios C, Devi LA, Gomes I (2005). Serotonin receptor activation leads to neurite outgrowth and neuronal survival. *Molecular Brain Research*.

[54] Meitzen J, Luoma JI, Stern CM, Mermelstein PG (2011). *β*1-Adrenergic receptors activate two distinct signaling pathways in striatal neurons. *Journal of Neurochemistry*.

[55] Azmitia EC (2001). Modern views on an ancient chemical: serotonin effects on cell proliferation, maturation, and apoptosis. *Brain Research Bulletin*.

[56] Milton NGN (2002). Anandamide and noladin ether prevent neurotoxicity of the human amyloid-*β* peptide. *Neuroscience Letters*.

[57] Pettifer KM, Kleywegt S, Bau CJ (2004). Guanosine protects SH-SY5Y cells against *β*-amyloid-induced apoptosis. *NeuroReport*.

[58] Wang Z, Zhang X, Wang H, Qi L, Lou Y (2007). Neuroprotective effects of icaritin against beta amyloid-induced neurotoxicity in primary cultured rat neuronal cells via estrogen-dependent pathway. *Neuroscience*.

[59] Wang H-Q, Sun X-B, Xu Y-X, Zhao H, Zhu Q-Y, Zhu C-Q (2010). Astaxanthin upregulates heme oxygenase-1 expression through ERK1/2 pathway and its protective effect against beta-amyloid-induced cytotoxicity in SH-SY5Y cells. *Brain Research*.

[60] Wang H-Q, Xu Y-X, Zhu C-Q (2011). Upregulation of heme oxygenase-1 by acteoside through ERK and PI3 K/Akt pathway confer neuroprotection against beta-amyloid-induced neurotoxicity. *Neurotoxicity Research*.

[61] Novaira HJ, Ng Y, Wolfe A, Radovick S (2009). Kisspeptin increases GnRH mRNA expression and secretion in GnRH secreting neuronal cell lines. *Molecular and Cellular Endocrinology*.

[62] Arai AC, Xia Y-F, Suzuki E, Kessler M, Civelli O, Nothacker H-P (2005). Cancer metastasis-suppressing peptide metastin upregulates excitatory synaptic transmission in hippocampal dentate granule cells. *Journal of Neurophysiology*.

[63] Choi SH, Aid S, Caracciolo L (2013). Cyclooxygenase-1 inhibition reduces amyloid pathology and improves memory deficits in a mouse model of Alzheimer's disease. *Journal of Neurochemistry*.

[64] Brenneis C, Maier TJ, Schmidt R (2006). Inhibition of prostaglandin E2 synthesis by SC-560 is independent of cyclooxygenase 1 inhibition. *The FASEB Journal*.

[65] Gulliver CE, Friend MA, King BJ, Robertson SM, Wilkins JF, Clayton EH (2013). Increased prostaglandin response to oxytocin in ewes fed a diet high in omega-6 polyunsaturated fatty acids. *Lipids*.

[66] Penrod LV, Allen RE, Rhoads ML, Limesand SW, Arns MJ (2013). Oxytocin stimulated release of PGF2*α* and its inhibition by a cyclooxygenase inhibitor and an oxytocin receptor antagonist from equine endometrial cultures. *Animal Reproduction Science*.

[67] Nakatani Y, Chin Y, Hara S, Kudo I (2007). Immediate prostaglandin E2 synthesis in rat 3Y1 fibroblasts following vasopressin V1a receptor stimulation. *Biochemical and Biophysical Research Communications*.

[68] Milton NGN, Hillhouse EW, Milton AS (1993). Does endogenous peripheral arginine vasopressin have a role in the febrile responses of conscious rabbits?. *Journal of Physiology*.

[69] Grassi D, Bellini MJ, Acaz-Fonseca E, Panzica G, Garcia-Segura LM (2013). Estradiol and testosterone regulate arginine-vasopressin expression in SH-sY5Y human female
neuroblastoma cells through estrogen receptors-*α* and -*β*. *Endocrinology*.

